# Increased Colonic Permeability and Lifestyles as Contributing Factors to Obesity and Liver Steatosis

**DOI:** 10.3390/nu12020564

**Published:** 2020-02-21

**Authors:** Domenica Maria Di Palo, Gabriella Garruti, Agostino Di Ciaula, Emilio Molina-Molina, Harshitha Shanmugam, Maria De Angelis, Piero Portincasa

**Affiliations:** 1 Clinica Medica “A. Murri”, Department of Biomedical Sciences and Human Oncology, University of Bari Aldo Moro, 70124 Bari, Italy; domenicamariadipalo@gmail.com (D.M.D.P.); agostinodiciaula@tiscali.it (A.D.C.); emilio.molinamolina@uniba.it (E.M.-M.); harshitha.shanmugam@uniba.it (H.S.); 2 Department of Soil, Plant and Food Sciences, University of Bari Aldo Moro, 70124 Bari, Italy; maria.deangelis@uniba.it; 3 Section of Endocrinology, Department of Emergency and Organ Transplantations, University of Bari “Aldo Moro” Medical School, 70124 Bari, Italy; gabriella.garruti@uniba.it

**Keywords:** body mass index, intestinal permeability, obesity, sugars, urinary recovery

## Abstract

Intestinal permeability (IP) is essential in maintaining gut-metabolic functions in health. An unequivocal evaluation of IP, as marker of intestinal barrier integrity, however, is missing in health and in several diseases. We aimed to assess IP in the whole gastrointestinal tract according to body mass index (BMI) and liver steatosis. In 120 patients (61F:59M; mean age 45 ± SEM 1.2 years, range: 18–75), IP was distinctively studied by urine recovery of orally administered sucrose (SO, stomach), lactulose/mannitol ratio (LA/MA, small intestine), and sucralose (SA, colon). By triple quadrupole mass-spectrometry and high-performance liquid chromatography, we measured urinary recovery of saccharide probes. Subjects were stratified according to BMI as normal weight, overweight, and obesity, and answered questionnaires regarding dietary habits and adherence to the Mediterranean Diet. Liver steatosis was assessed by ultrasonography. IP at every gastrointestinal tract was similar in both sexes and decreased with age. Stomach and small intestinal permeability did not differ according to BMI. Colonic permeability increased with BMI, waist, neck, and hip circumferences and was significantly higher in obese than in lean subjects. As determined by logistic regression, the odds ratio (OR) of BMI increment was significantly higher in subjects in the highest tertile of sucralose excretion, also after adjusting for age and consumption of junk food. The presence of liver steatosis was associated with increased colonic permeability. Patients with lower score of adherence to Mediterranean diet had a higher score of ‘junk food’. Intestinal permeability tended to increase in subjects with a lower adherence to Mediterranean diet. In conclusion, colonic (but not stomach and small intestinal) permeability seems to be linked to obesity and liver steatosis independently from dietary habits, age, and physical activity. The exact role of these last factors, however, requires specific studies focusing on intestinal permeability. Results should pave the way to both primary prevention measures and new therapeutic strategies in metabolic and liver diseases.

## 1. Introduction

Intestinal permeability (IP) is dependent on the structure and function of the intestinal barrier [1,2]. The gut barrier integrity is the result of ongoing equilibrium and crosstalk involving the microbiome, the mucus, the enterocytes, the gut immune system, and the gut–vascular barrier. In this context, a link exists between antigenic/toxic molecules in the gut lumen and the systemic status in health and disease [3,4,5,6]. The derangement of IP can originate from conditions involving chronic local and systemic inflammation, intestinal dysbiosis, and also metabolic changes occurring during onset and development of metabolic syndrome, obesity, and liver steatosis [7]. In particular, markers of IP, such as circulating zonulin, increase with obesity-associated insulin resistance [8]. Increased IP occurs also in obese women with metabolic syndrome [9], and abnormal IP may develop in association with changes of gut microbiota, a source of endotoxins whose increase in plasma is related to obesity and insulin resistance [10]. Further evidences point to a link between increased IP and pathogenic aspects of non-alcoholic fatty liver disease (NAFLD) [11,12].

**Figure 1:**
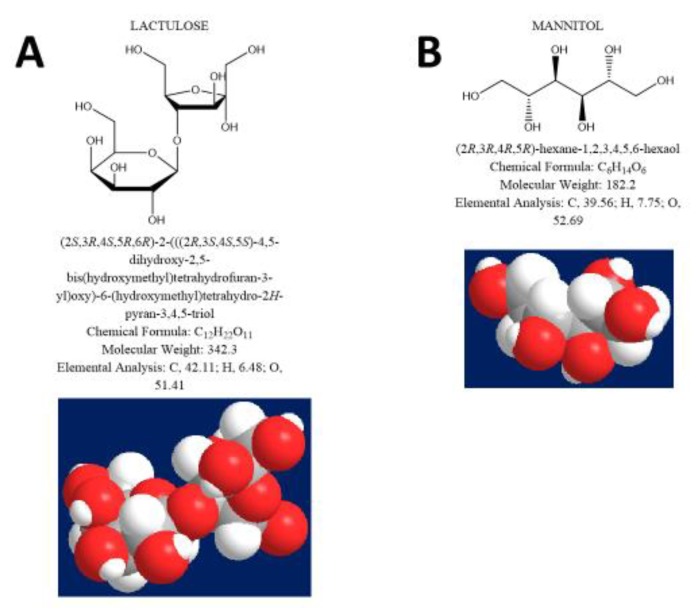

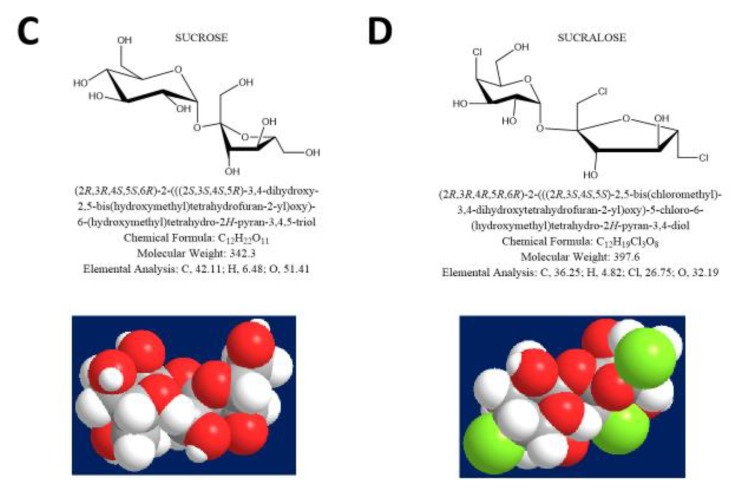
Chemical and 3-D formulas of the sugar probes used for the study of intestinal permeability at different tracts: Lactulose (**A**), mannitol (**B**), sucrose (**C**) and sucralose (**D**).

A measure of IP [13,14] is based on the detection of specific probes, either in blood or in urine [1]. Non-metabolizable sugars, chromium-labelled EDTA (^51^Cr-EDTA), and polyethylene glycols (PEG) are typical marker probes [15]. In humans, IP at the level of duodenum and small intestine is measurable after oral administration of large oligosaccharide probes, such as lactulose (LA) (m.w. 342.3), combined with a small sugar such as mannitol (MA) (m.w. 182.2) [16]. Both these probes are equally affected by gastrointestinal dilution, motility, bacterial degradation, and renal function. Therefore, the LA/MA ratio is corrected for possible confounding factors [3], as a marker of paracellular permeability in the small intestine [14,17]. Sucrose (SO) (m.w. 342.3), by contrast, tests the permeability of the upper intestinal tract, since it is degraded by duodenal sucrose [14]. Sucralose (SA) (m.w. 397.6) is an artificial sugar poorly absorbed in the human intestine, not degraded by intestinal bacteria, and useful in assessing the permeability of the entire intestinal tract [3] and colon [18] (Figure 1A–D). 

Intestinal barrier function has a relevant role in health and disease [1]. Due to the intrinsic length and surface of the intestine, however, more studies should focus on the intestinal barrier integrity at different levels in the gastrointestinal tract. This approach is of key importance when considering the raising worldwide epidemics of obesity, metabolic syndrome, and liver steatosis [19,20], and the possibility to start targeting the intestine by several pharmacological and non-pharmacological agents [7].

Lifestyle habits can have an effect on intestinal permeability, especially when considering energy-dense food products, physical exercise, environmental conditions, or drugs, among others [1]. In this respect, the Mediterranean Diet can modulate the composition and diversity of gut microbiota. The Mediterranean Diet is traditionally consumed by people living by the Mediterranean Sea. This diet emphasizes on a high intake of vegetables, fruits, nuts, and cereals; moderate intake of fish and poultry; low intake of dairy, meat products, and sweets; with olive oil as the principle source of fat [21]. Extreme physical exercise can also induce intestinal damage and increase intestinal permeability [22].

Here, we studied the pan-enteric IP (stomach, small intestine, and colon) with respect to size and fat distribution, as well as the presence of liver steatosis (i.e., NAFLD, recently re-termed metabolic associated fatty liver disease, MAFLD [23]). We also examined the impact of adherence to the more protective Mediterranean Diet regimen and further stratified the study groups by age and gender.

## 2. Materials and Methods 

### 2.1. Subjects

We consecutively enrolled 205 subjects from the outpatient clinic at the Division of Internal Medicine of the Regional Hospital “Policlinico”, University Medical School in the Apulian city of Bari. The hospital serves the metropolitan area of Bari (323,400 inhabitants). Excluded were 85 subjects with celiac disease, Familial Mediterranean Fever, inflammatory bowel diseases, irritable bowel syndrome, constipation, gastrointestinal neoplasms, and gallstones. Further exclusion criteria were presence of structural abnormality of the gastrointestinal tract, overt diabetes mellitus or anti-diabetic therapy, major abdominal surgery, biliary duct obstructions, positive stool culture for common pathogenic bacteria, mental illness, concomitant immunological, hematological or other neoplastic disease, liver cirrhosis, and severe heart failure (NYHA class II-IV). Drug abuse was excluded as well as alcohol abuse (i.e., ongoing or recent alcohol consumption >21 standard drinks on average per week in men and > 14 standard drinks on average per week in women [24]).

During the two weeks before the study, medications such as non-steroidal anti-inflammatory drugs, antibiotics, fibers, or probiotics were forbidden. Subjects taking concomitant treatments including antispasmodic, anticoagulants, anticholinergics, pro-kinetics and osmotic laxative, antidepressant, or anxiolytic drugs were excluded. The final group included 120 subjects (61 females and 59 males; mean age 45 ± SEM 1.2 years, range 18–75). 

The local Ethics Committee of University Hospital Policlinico in Bari approved this protocol (study number 5408, protocol number 0013869; approved by AOUCPG23/COMET/P on 7th July 2017). The study was performed in accordance to the guidelines of the Declaration of Helsinki. Before the study, all patients gave full written informed consent to allow all authors to access the study data and use them for research purpose only.

### 2.2. Anthropometric Measurements

Anthropometric variables included in the study were body weight (kg), height (cm), and body mass index (BMI), calculated as the Quetelet’s index, i.e., kilograms divided by meter squared (kg/m^2^) [25,26]. BMI ranging from 18.5 to 24.9 kg/m^2^ defined normal weight subjects, while a BMI ranging from 25.0 to 29.9 kg/m^2^ and >30 kg/m^2^ defined overweight and obese subjects, respectively. We measured the waist, hip, and neck circumferences by using a non-stretching tape. In each subject, waist was measured at the superior border of the iliac crest according to the indications of the Revised National Cholesterol Education Programme-Adult Treatment Panel III (R-ATPIII), and between the iliac crest and the lower border of ribs according to the indications of the International Diabetes Federation (IDF). According to R-ATPIII, cut-off values were 88 cm and 102 cm for females and males, respectively. For IDF, cut-off values were 80 cm and 94 cm for Caucasian females and males, respectively. For neck circumference, cut-off values were 34 cm and 40 cm in females and males, respectively [27,28,29,30]. The cut-off value for hip circumference was <102 cm. 

### 2.3. Questionnaires

All patients answered a custom-designed questionnaire (‘MedStyle’) [31], which assessed several food products, both qualitatively and quantitatively (including ‘fast-food’ products), frequency of physical exercise (from zero to seven times per week), alcohol consumption, carbonated drinks, sweets, and salt. Also, each subject had to watch detailed images representing the different types of foods and drinks to estimate the frequency, quality, and quantity (i.e., small, medium, and large portion) of food consumed, according to a validated version of Winfood^®^ software (Medimatica, Colonnella, Teramo Italy) [32]. A ‘Junk Score’ was also calculated based on consumption of 12 high fat, high-sugar food products. Additionally, we assessed adherence to Mediterranean Diet based on an 18-point scale [33]. We classified adherence as “inadequate” in subjects with a score of less than 10 points. 

### 2.4. Measurement of Intestinal Permeability

To measure intestinal permeability (IP), four saccharide probes were administered orally and simultaneously [18]. Stomach permeability was assessed by administering 20 g of SO, small intestine permeability was assessed by administering LA (5 g) and MA (1 g), and colonic permeability with 1 g of SA. The procedure followed the guidelines of the manufacturer (AB Analitica s.r.l., Padova, Italy).

One week before the test, subjects had to avoid laxatives, prebiotics, probiotics, and synthetic sugars. The day before the test, each subject received dietary indications to abstain from consumption of chocolate, sweets, chewing gum, fruit juices, fresh or packaged fruit, dietetic products, ice cream, and alcohol, to avoid any potential interference with the test. Additionally, each subject could only consume grilled meat or fish and had to drink 1 L of water before bedtime. A urinary sample was collected in the morning of the test, after an overnight fast. After that, the test sugars dissolved in 250 mL of tap water were orally administered. Thereafter, urine was collected every hour over six hours in a sterile container containing chlorhexidine. During the whole test, subjects had to remain fasted. IP was calculated from the 6-h urinary recovery. Two samples for patients were analyzed by urinary plasma chromatography/mass spectrometry (UPLC-MS/MS, AB Analitica, Padua, Italy). The fraction of the excreted sugars was calculated based on the quantity of sugar ingested by the patients and expressed as a percentage value, according to the manufacturer. Their presence in urine, in different amounts compared to the expected, indicated impaired permeability of the stomach, small intestine, or colon. Normal values for permeability test were expressed as percent recovery (i.e., <0.15% for SO, <0.03 for LA/MA ratio, and <1.5% for SA). 

### 2.5. Measurement of Liver Steatosis

Liver steatosis was measured with the ‘Hitachi Noblus-E echocolordoppler’ (Hitachi Medical, Tokyo, Japan) and Logiq E9 (GE, Healthcare) ultrasound equipment, using a 3.5 MHz convex probe. In particular, kidney cortex echogenicity (control) was compared with the echogenicity of the liver parenchyma. Ultrasonography reliably detects a hyperechoic texture or a bright liver upon diffuse fatty infiltration [34]. This finding becomes a sensitive marker of liver steatosis (ranging from grade 0 = absent to grade 3 = severe steatosis) [35,36,37,38,39,40]. 

### 2.6. Measurement of Liver Fibrosis

The degree of liver fibrosis was non-invasively assessed by acoustic radiation force impulse (ARFI) imaging, using a 3.5 MHz convex probe and validated cut-off values [41]. 

### 2.7. Statistical analysis

Statistical analyses were performed using the NCSS statistical software (‘NCSS10′ Statistical Software 2013, NCSS, LLC. Kaysville, Utah, USA [42,43] (www.ncss.com/software/ncss). Data represent means ± standard error of the mean (SEM), medians, range, or percentages, as appropriate. Data between groups were compared by the Student’s t-test (two groups) or by one-way analysis of variance (ANOVA) followed by Tukey–Kramer Multiple-Comparison Test. For a one-way ANOVA comparing three groups, 21 is the sample size needed in each group to obtain a power of 0.80, when the effect size is large and a significance level of 0.05 is employed (calculated with R software version 3.4.1, package “pwr”). Proportions were compared by the Chi-square test. Levels of excreted sugars were categorized according to their tertiles. To calculate odds ratios (OR) and confidence intervals (CI) for BMI associated with sugar excretion levels, separate logistic regression models were fitted, with BMI value as the dependent variable and tertiles of sugar excretion as independent variables. The models were adjusted according to possible confounders. Models were fitted using R software version 3.1.1 (The R Foundation for Statistical Computing, Vienna, Austria). Results were considered significant at the 5 % critical level (*p* < 0.05). ‘SigmaPlot’ v. 14.0 was used to represent data as graphs.

## 3. Results

### 3.1. Clinical Features

Table 1 depicts the clinical features of the three study groups according to body mass index (BMI), i.e., normal weight, overweight, and obese. 

Groups were gender-matched except for the prevalence of males in the overweight group. Age was similar across the three groups, with 18% of women and 12% of men younger than 30, 72% of women and 80% of men ranging between 30 and 64 years, and 10% of women and 8% of men older or equal to 65 years.

**Figure 2:**
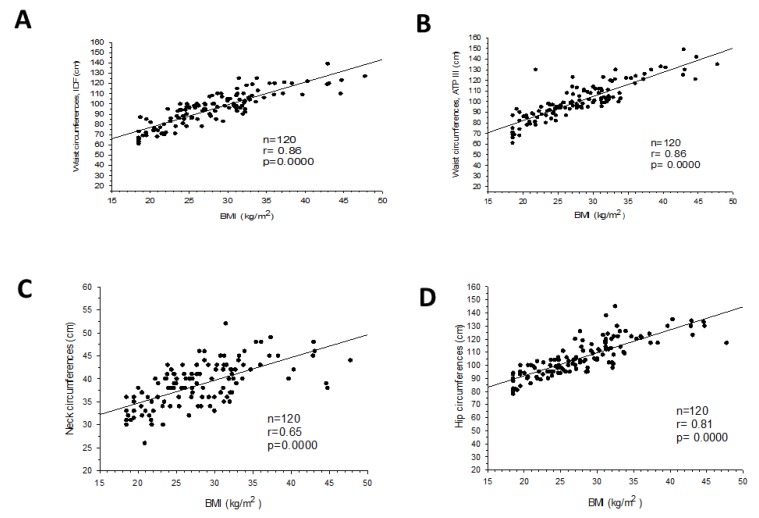
Correlation between body mass index (BMI) and waist circumference according to: International Diabetes Federation (IDF) (**A**), Adult Treatment Panel III (ATPIII) (**B**), neck circumference (**C**), and hip circumference (**D**).

BMI, waist, hip, and neck circumferences progressively increased from normal weight to overweight and to obese subjects, in both genders. Waist (IDF and ATPIII), neck, and hip circumferences increased significantly with BMI (Figure 2A–D).

Liver steatosis was detected in 69 (57.5%) subjects, of which 36 (52%) were males. The prevalence of liver steatosis increased from 4% in normal weight subjects to 77%, and to 98% in overweight and obese subjects, respectively. This increasing prevalence paralleled the increase in the degree of liver steatosis at ultrasonography. Furthermore, all subjects were reported to have, on average, absent or very mild liver fibrosis (F0/F1).

### 3.2. Intestinal Permeability and Age

**Figure 3:**
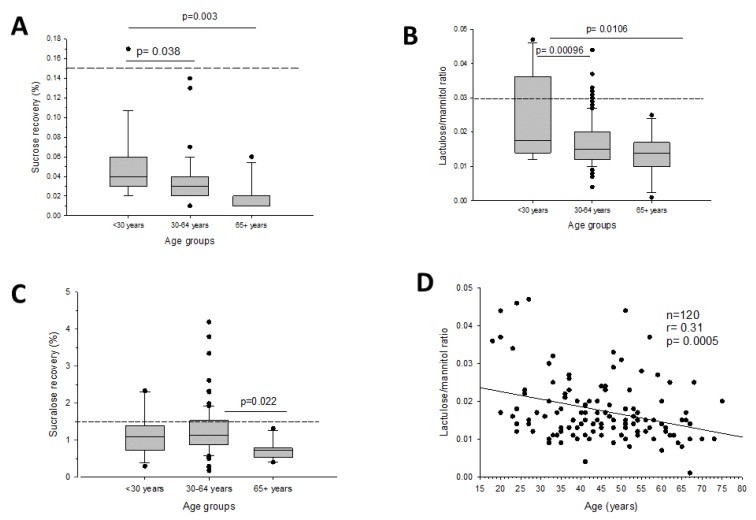
Intestinal permeability by tract and age groups: Stomach permeability (**A**), small intestinal permeability (**B**), colonic permeability (**C**), and correlation between small intestinal permeability and age (**D**).

Gastrointestinal permeability changed between age groups at every tract (i.e., < 30 years; 30–64 years; and ≥ 65 years). Stomach and small intestinal permeability decreased with age (Figure 3A–B). The same findings were obtained at the colonic level, especially in the ≥65 years subgroup (Figure 3C). Interestingly, the strongest evidence was found when small intestinal permeability was plotted against increasing age, a finding persisting in both sexes (Figure 3D).

### 3.3. Intestinal Permeability and Anthropometric Markers

Recovery of the four-saccharide probes at different gastrointestinal levels according to BMI appears in Table 2. 

Sucrose recovery (stomach) and lactulose/mannitol ratio (small intestine) were similar in normal weight, overweight, and obese subjects. 

**Figure 4:**
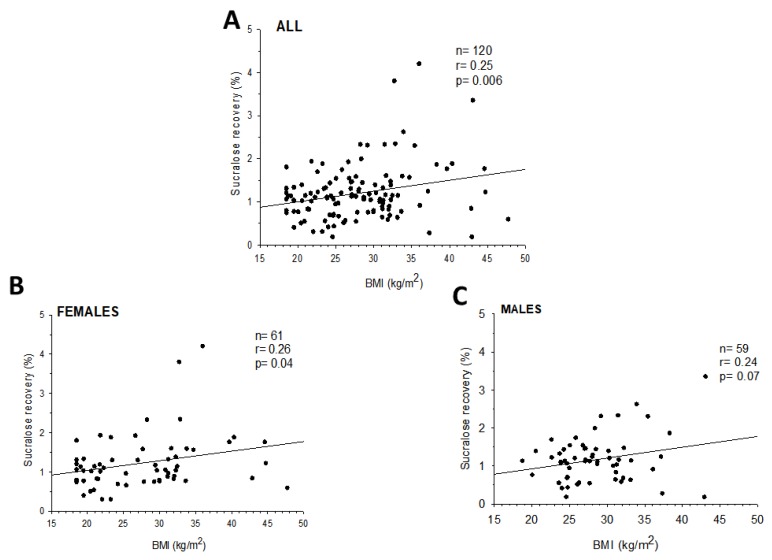
Correlation between sucralose recovery and BMI in all subjects (**A**), females (**B**), and males (**C**).

By contrast, sucralose recovery (colonic permeability) tended to increase in overweight subjects and increased significantly in obese, as compared to normal-weight subjects (*p* = 0.014). The prevalence of abnormal colonic permeability was higher in both overweight and obese than in normal weight subjects, and sucralose recovery increased significantly with BMI overall, in females (*p* = 0.04), and tended to increase also in males (*p* = 0.07, Figure 4).

**Figure 5:**
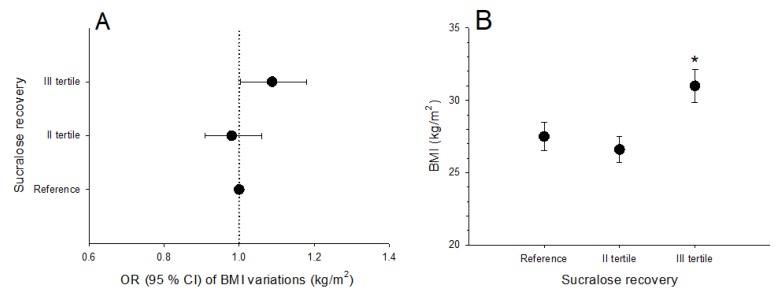
(**A**) shows odds ratios and 95 % confidence intervals of BMI variations (kg/m^2^) in subjects grouped according to tertiles of sucralose urinary excretion. Values were calculated by logistic regression models, with BMI as the dependent variable and tertiles of sucralose recovery as the independent variable, after adjusting for covariates (age, junk score). (**B**) indicates average BMI (kg/m^2^) in subjects grouped according to tertiles of sucralose recovery. Data are mean ± SE. *p = 0.02 vs. reference group and subjects in the second tertile (ANOVA followed by Fisher’s LSD multiple comparison test).

In the whole population, the OR of BMI increment according to sucralose excretion was significantly higher in subjects in the highest tertile (sucralose excretion >1.45%; OR 1.085 [95% CI: 1.006, 1.17]), than in those in the reference and in the second tertile. The result persisted (1.06 [95% CI: 1.003, 1.18]) after adjusting for age and consumption of junk food (junk score), considered as covariates (Figure 5A). The average BMI was higher in subjects in the highest tertile of sucralose excretion, as compared to those in the reference group and in the second tertile (Figure 5B).

**Figure 6:**
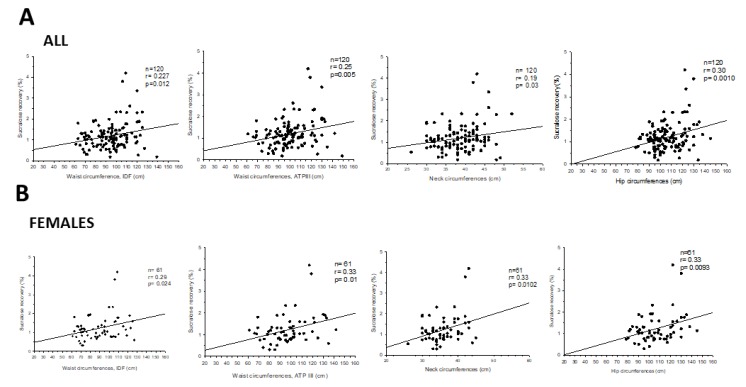
Correlation between sucralose recovery and waist, neck, and hip circumferences in: All subjects (**A**) and in females (**B**).

Colonic permeability also increased with waist, neck, and hip circumferences in females (Figure 6), but not in males.

### 3.4. Liver Steatosis and Intestinal Permeability 

Data on gender, age, BMI, waist, hip and neck circumferences, and intestinal permeability according to liver steatosis groups (without vs. with) appear in Table 3. 

Gender distribution was similar in the two groups, but subjects with liver steatosis were significantly older and heavier than subjects without liver steatosis. In accordance with these findings, waist, hip, and neck circumferences were higher in subjects with liver steatosis than in those without liver steatosis, regardless of gender. Data on recovery of the four-saccharide probes according to liver steatosis show that means of sucrose recovery and lactulose/mannitol ratio were comparable between groups. However, sucralose recovery (i.e., colonic permeability) increased significantly in subjects with liver steatosis than in those without liver steatosis. 

### 3.5. Intestinal Permeability and Diet

The majority of enrolled subjects (93%) had adequate adherence to Mediterranean diet. The prevalence of inadequate adherence was similar between lean and overweight subjects (16% and 20 %, respectively), and slightly higher in obese subjects (31 %) (Table 1).

Table 4 depicts the profile of two groups according to adherence to Mediterranean Diet, compared for anthropometric features, ‘junk food’ intake, and intestinal permeability. 

**Figure 7:**
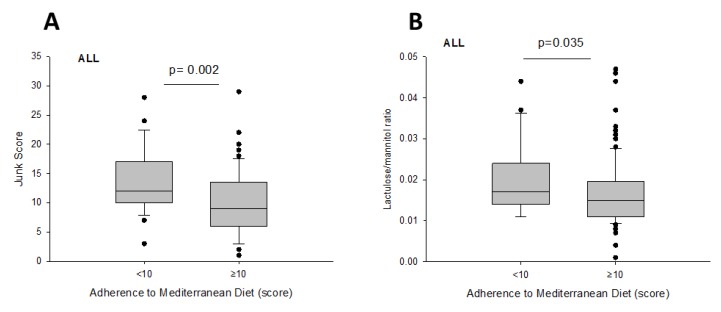
Differences in ‘Junk Score’ (**A**) and small intestinal permeability (**B**) according to adherence to Mediterranean Diet.

Subjects with a score ≥ 10 points (i.e., adequate adherence) were older, consumed less ‘junk food’, and had slightly lower small intestinal permeability, compared with subjects with inadequate adherence (Figure 7A,B). All other parameters were comparable between both groups.

### 3.6. Intestinal Permeability and Physical Exercise

Among the enrolled subjects, 45 % (of which 48% were lean, 32% overweight, and 20% obese) performed physical exercise at least once weekly. Physical exercise frequency was unchanged when classifying subjects according to their BMI, liver steatosis, or obesity condition. However, females exercised significantly less than males (1.1 ± 0.2 vs. 1.8 ± 0.3 times a week respectively, *p* = 0.015). Intestinal permeability was unaffected by physical exercise. 

## 4. Discussion

The present study provides, for the first time, information deriving from a combined and extensive analyses of the permeability of the gastrointestinal tract with respect to metabolic profiles, in a human cohort ranging from healthy, to overweight and obese people. We particularly explored aspects of pan-enteric intestinal permeability in relation to age, anthropometric variables, lifestyles, obesity, and liver steatosis. The non-invasive “four-sugar” urinary recovery test allows the simultaneous study of the stomach, small intestinal, and colonic permeability in health and disease. Exploring gastrointestinal permeability in different metabolic abnormalities can develop the understanding of further pathogenic mechanisms underlying chronic extra-intestinal diseases, and eventually, it could support the treatment in primary and secondary prevention. The results of the present study point to significant topographic differences of intestinal permeability during gut-liver metabolic abnormalities. 

Growing evidences document how the gastrointestinal tract, beyond digestion of nutrients, plays a crucial role in maintaining systemic homeostasis, through its function of barrier between intraluminal contents and systemic circulation. The gut barrier integrity is therefore essential to maintain the intestinal homeostasis, which is a result of a continuous interplay between nutrients, bacterial metabolism, trans-epithelial absorption, and local inflammation [4,5,6].

The so-called gut-vascular unit includes the intestinal endothelium associated with pericytes and enteric glial cells, tight junctions, and adherent junctions (permeable to most of the small nutrients) [44,45,46]. This barrier selectively absorbs nutrients and molecules, and prevents the abnormal translocation of bacteria and/or microbial components. The intestinal barrier is typically disrupted in conditions like celiac disease [44], and anchylosing spondylitis [47]. In addition, human studies point to a role for increased intestinal permeability, disrupted tight junctions, and small intestinal bacterial overgrowth (SIBO) in NAFLD (MAFLD [23]) patients [48]. In principle, alterations of the gastrointestinal permeability could also increase the risk of systemic diseases, such as obesity [49] and diabetes [50,51]. 

Although the permeability of the stomach and the small intestine was independent from BMI, we found that aging does influence intestinal permeability. In fact, stomach and small intestinal permeability decreased with age, while still falling within the normal range. This finding also occurred in subjects aged over or equal to 65 years, with respect to colonic permeability. 

This is in line with previous studies on decreased small intestinal permeability alongside age, likely due to an age-related loss of renal function [52]. In addition, gut microbiota could be involved. Gut microbiota is responsive to lifestyles, age, birth mode, and contributes to maintaining the mucosal barrier [53]. Gut microbiome also contributes to keep a healthy function of the epithelial intestinal cells and of the immune system [54], producing local peptides with antimicrobial function and immunoglobulins [55,56,57]. Elderly and younger individuals display differences in gut microbiome composition [58]. Such age-related changes at various gastrointestinal levels might therefore also govern the changes in intestinal permeability during aging “per se”, and/or be sign of progressive atrophic changes in the intestinal barrier. The present study was not designed to assess the profile of gut microbiota in the enrolled subjects or microbiota-related metabolome. These aspects should be specifically explored in future studies linking the role of gut microbiome and bacterial products, with local effects on the dynamics of epithelial barriers, and therefore intestinal permeability. Notably, in irritable bowel syndrome (IBS) patients, we recently found that oral administration of *Bifidobacterium longum* BB536 and *Lactobacillus rhamnosus* HN001 with vitamin B6 improved symptoms, severity of disease, while restoring intestinal permeability, modifying the composition of gut microbiota [59].

As expected, we confirmed that the anthropometric features in our study increased with BMI in both sexes. However, we found that obesity was associated with increased colonic permeability. This finding occurs without evidence of altered stomach and small intestinal permeability. Although the number of subjects in the three subgroups was relatively low, a significant difference in colonic permeability between subjects belonging to different BMI categories (i.e., normal weight, overweight, and obese) was evident. At variance with data regarding sucralose recovery (i.e., colonic permeability), mean values on sucrose recovery (i.e., stomach permeability) and lactulose/mannitol ratio (i.e., small intestinal permeability) were very similar among groups, without evidence of possible inter-group trends.

Obese patients are predisposed to presence of a low-grade systemic and metabolic inflammation, due to increased visceral fat [60]. During mucosal inflammation, changes in the homeostasis of the intestinal barrier occurs, causing damage to the structure and further modification of the apical junctions. At this stage, intestinal permeability may increase [61], causing the translocation of harmful and pathogenic substances, microorganisms, and/or molecules from the intestinal lumen into the bloodstream [62], such as the lipopolysaccharide of gram-negative bacteria. Clinically, the colon is a 1.5-m-long organ responsible for reabsorption of water at the right side of the intestine, and deposit of material and microbiota at its left side. The microbiota reaches a maximum concentration in the colon of 10^12^ CFU/mL, playing an important role in the formation of vitamins to the biotransformation of nutrients with production of metabolites. Likely, the data related to the increase in colonic permeability could be an indicative marker of qualitative or quantitative dysbiosis, as found in the literature dealing with obese individuals [10]. However, the link between human obesity, intestinal permeability modification, changes in gut microbiota, and inflammation is poorly documented. 

Our data allow us to speculate that impaired barrier function is associated with increased local chronic low-grade inflammation, which is a common feature in metabolic syndrome and/or central obesity. Further studies are required to establish whether chronic low-grade inflammation antedates or is a consequence of metabolic changes, or rather represents a casual association [63]. 

Obesity is influenced by various factors such as dietary behaviors, specific lifestyles, and is associated with reduced microbial diversity [64]. Obesity is also a principal feature of metabolic syndrome and anthropometrics measurements (a simple and useful method for predicting metabolic syndrome [65,66]), which are further increased in obese subjects with respect to normal and overweight subjects. 

Of note, as shown in our study by a logistic regression analysis, the altered colonic permeability in obese subjects was still present when considering age as a confounding factor. According to our results, in fact, sucralose excretion was a predictor of increased BMI, regardless of age and ‘junk food’.

An original finding in the present study is that 98 % of obese subjects had liver steatosis and increased colonic permeability as compared to subjects without liver steatosis. This result highlights the involvement of the gut–liver axis in the presence of intestinal dysbiosis. A recent study in C57BL/6J mice fed with a high-fat diet or methionine choline-deficient diet points to the possibility that microbiota-driven gut vascular barrier disruption is a prerequisite for NASH [67]. Indeed, one of the factors involved in the genesis and maintenance of hepatic steatosis is the influx of bacterial products, leading to activation of the hepatic systems that initiate damage with TNF-α release or reduction of cell function by Kupffer cells. 

Factors such as disrupted tight junction of the enterocytes, dysbiosis, and/or SIBO might contribute to initiate the steatogenic process [48,68]. A recent meta-analysis showed that patients with NAFLD more frequently exhibit altered intestinal permeability [69]. Children with NAFLD showed a positive correlation between altered intestinal permeability, liver inflammation, and liver fibrosis [70]. SIBO can develop in NAFLD patients [48,71,72,73,74], along with dysbiosis [75]. Abnormal intestinal permeability might therefore be involved in this scenario [76], acting as a factor leading to liver inflammation and fibrosis. A previous trial compared 22 patients with biopsy-proven NASH and 23 controls subjects. The authors assessed SIBO by combining (14)C-D-xylose and lactulose breath test, intestinal permeability by a dual lactulose-rhamnose sugar test, serum endotoxin levels by limulus amoebocyte lysate assay, and TNF-α levels by ELISA. Results showed that 50 % of patients and 22 % of controls had SIBO. Whereas intestinal permeability and serum endotoxin levels were similar in the two groups, TNF-α levels were significantly higher in patients than controls (14.2 and 7.5 pg/mL, respectively) [77]. 

Another study compared patients with biopsy-proven NAFLD (*n* = 35), patients with untreated celiac disease (*n* = 27), as a model of intestinal hyper-permeability, and healthy subjects (*n* = 24) [48]. The authors used glucose hydrogen breath testing to diagnose SIBO, and urinary excretion of (51) Cr-ethylene diamine tetraacetate ((51) Cr-EDTA) test to diagnose intestinal permeability. The authors also used immune-histochemical analysis of zona occludens-1 (ZO-1) expression in duodenal biopsy specimens to diagnose the integrity of tight junctions within the gut. Orally administered ^51^Cr-EDTA is not metabolized and is poorly absorbed (1–3%) from the gastrointestinal tract. In the presence of TJ disruption, ^51^Cr-EDTA crosses the intestinal barrier through the paracellular pathway [78,79,80]. NAFLD patients had significantly increased intestinal permeability and SIBO compared to control [48]. ^51^Cr-EDTA excretion levels and SIBO prevalence increased with liver steatosis. In addition, by duodenal histology, NAFLD patients had reduced ZO-1 expression. Increased intestinal permeability and SIBO were independent of the severity of liver inflammation, liver fibrosis, and NASH.

NAFLD children had increased ratio in urinary excretion of orally administered lactulose and mannitol (L/M ratio), as a marker of intestinal permeability [70,77,81]. L/M ratio further increased in NASH patients. The increased lipopolysaccharides pointed to bacterial translocation while the extent of hepatic inflammation and fibrosis was proportional to the degree of intestinal permeability. However, colonic permeability was not studied [70].

The possibility of intestinal dysbiosis, including SIBO, was not ruled out in this study, but we did find deranged intestinal permeability in a subset of subjects. All major predisposing factors to SIBO, including intestinal and systemic inflammations, aging, hypo-achlorhydria due to chronic autoimmune atrophic gastritis or prolonged use of proton pump inhibitors, and use of antibiotics were absent in the study groups. In addition, both unspecific (e.g., bloating, abdominal discomfort, flatulence) and more specific generalized symptoms (e.g., malabsorption and nutrient deficiency with diarrhea, steatorrhea, and weight loss) for SIBO were absent in all enrolled subjects. Generally, the overall number of patients with SIBO is low [31,82,83,84], as assessed by lactulose/glucose hydrogen breath test [85]. Other methods were not employed to diagnose SIBO (i.e., at least 10^5^ colony-forming units (CFU) per milliliter (mL) of jejunal aspirate) [86] due to the invasive characteristics of the test. Nevertheless, the possibility exists that qualitative and/or quantitative changes of intestinal microbiota occur in terms of temporal (age) and/or topographical (proximal vs. distal intestine) distribution of intestinal bacteria. An example derives from a recent study in NAFLD patients displaying elevated localization of lipopolysaccharides in hepatocytes, partly dependent on Escherichia Coli in intestine [87]. These changes can still occur in obese subjects with liver steatosis and remain under-diagnosed but still contributing to selective changes of the intestinal permeability. Sedentary behaviors and their impact on gut microbiota may play a role in this respect [88,89].

Subjects enrolled in the present study showed, overall, an adequate compliance to Mediterranean diet. Nevertheless, obese patients had a slightly but still lower adherence to Mediterranean diet, while subjects less compliant to Mediterranean diet consumed more ‘junk food’. Of note, subjects with inadequate adherence to Mediterranean diet had slightly but significantly increased small intestinal permeability. Changes were limited to the small intestine and might reflect dietary-microbiome-driven changes, without a clear effect on stomach and colonic permeability.

These observations require larger and prospective studies to discern the role of Mediterranean Diet on the prevention and management of metabolic and associated diseases [90,91]. Furthermore, the questionnaire we used in our study was aimed to assess the timing but not the load (i.e., intensity and volume) of exercise performance in our subjects. This aspect should be relevant [22], and needs further exploration in order to provide a comprehensive picture about the relationships between exercise intensity, gastrointestinal permeability, and metabolic disorders [89].

Further studies also urge to evaluate the role of the gut microbiota on the intestinal permeability to allow both primary prevention measures and new therapeutic strategies in metabolic and liver diseases.

## 5. Conclusions

In conclusion, results from the present study highlight the existence of an association between colonic (but not stomach and small intestinal) permeability, obesity, and liver steatosis. This link could be independent from dietary models, age, and physical activity, although the exact role of these last factors still needs to be better determined in larger populations and by specific study designs. Further studies must evaluate the possibility of modulating colonic permeability to allow both primary prevention measures and new therapeutic strategies in metabolic and liver diseases.

## Figures and Tables

**Table 1:** Clinical characteristics of the study groups.

	Normal Weight	Overweight	Obese
Number	45	30	45
Males (%): Females (%)	18 (40 %):27 (60 %)	20 (67 %):10 (33 %) *	21 (47 %):24 (53 %)
Age (years)	42 ± 2	48 ± 2	45 ± 2
BMI (kg/m^2^)	21.7 ± 0.33	27.4 ± 0.24 *	34.6 ± 0.7 *^,#^
Waist circumference (cm) ^1^			
Males	89.4 ± 2	96.8 ± 1.9 *	111.3 ± 1.9 *^,#^
Females	72.9 ± 1.8	92.9 ± 3 *	107.2 ± 1.9 *^,#^
Waist circumference (cm) ^2^			
Males	89.4 ± 2.3	102 ± 2.2 *	116.5 ± 2.17 *^,#^
Females	82.7 ± 2.4	99.1 ± 3.9 *	112.8 ± 2.6 *^,#^
Hip circumference (cm)	94 ± 1.3	104.9 ± 1.6 *	118.7 ± 1.3 *^,#^
Neck circumference (cm)			
Males	38.8 ± 0.6	40.7 ± 0.6	44.8 ± 0.6 *^,#^
Females	32.7 ± 0.6	35.5 ± 0.9 *	39.3 ± 0.6 *^,#^
Liver steatosis, prevalence (%)	2 (4%)	23 (77%) *	44 (98%) *^,#^
Degree of liver steatosis	0 ± 0.08	0.9 ± 0.02 *	1.8 ± 0.08 *^,#^
Inadequate adherence to Mediterranean Diet, prevalence (%)	7 (16%)	6 (20%)	14 (31%) *

Data expressed as mean ± SEM. Differences tested by one-way ANOVA or Chi-square test. Significance levels: * *p* < 0.05 vs. normal weight; ^#^ *p* < 0.05 vs. overweight. ^1^ According to International Diabetes Federation (IDF); ^2^ according to Adult Treatment Panel III (ATPIII).

**Table 2:** Intestinal permeability at different gastrointestinal tracts, according to BMI.

	Normal Weight	Overweight	Obese
Number	45	30	45
Stomach (sucrose recovery, %)	0.03 ± 0.003	0.03 ± 0.003	0.04 ± 0.004
Median	0.03	0.03	0.03
Range	0.01–0.13	0.01–0.1	0.01–0.17
Small intestine (lactulose/mannitol, ratio)	0.018 ± 0.001	0.018 ± 0.002	0.02 ± 0.001
Median	0.016	0.014	0.02
Range	0.007–0.046	0.004–0.047	0.001–0.044
Colon (sucralose recovery, %)	0.99 ± 0.06	1.24 ± 0.09	1.37 ± 0.13 *
Median	1.06	1.18	1.14
Range	0.18–1.93	0.51–2.33	0.18–4.2

Data expressed as mean ± SEM. Differences tested by one-way ANOVA. Significance levels: * *p* < 0.05 vs. normal weight.

**Table 3:** Anthropometric features according to liver steatosis and intestinal permeability.

	Without Liver Steatosis	With Liver Steatosis
Number	51	69
Males (%): Females (%)	23 (45%):28 (55%)	36 (52%):33 (48%)
Age (years)	41.7 ± 2.1	47.2 ± 1.4 *
BMI (kg/m^2^)	22.5 ± 0.4	31.9 ± 0.6 *
BMI subgroups		
Normal weight, prevalence (%)	43 (84%)	2 (3%) *
Overweight, prevalence (%)	7 (14%)	23 (33%) *
Obese, prevalence (%)	1 (2%)	44 (64%) *
Waist circumference (cm) ^1^		
Males	90 ± 1.4	105.9 ± 1.9 *
Females	75 ± 2.1	102.1 ± 2.3 *
Waist circumference (cm) ^2^		
Males	90.9 ± 1.6	111.2 ± 2.1 *
Females	82.0 ± 1.9	110.1 ± 2.4 *
Hip circumference (cm)	95.3 ± 1.1	113.9 ± 1.4 *
Neck circumference (cm)		
Males	39.3 ± 0.5	43.1 ± 0.6 *
Females	32.9 ± 0.5	38.2 ± 0.6 *
Stomach (sucrose recovery, %)	0.04 ± 0.003	0.04 ± 0.003
Median	0.03	0.03
Range	0.01–0.13	0.01–0.17
Small intestine (lactulose/mannitol, ratio)	0.02 ± 0.001	0.02 ± 0.001
Median	0.016	0.015
Range	0.007–0.047	0.001–0.044
Colon (sucralose recovery, %)	0.99 ± 0.06	1.35 ± 0.09 *
Median	1.06	1.17
Range	0.18–1.88	0.18–4.2

Data expressed as mean ± SEM. Differences tested by Mann–Whitney U test. Significance levels: * *p* < 0.05 vs. without liver steatosis. ^1^ According to International Diabetes Federation (IDF); ^2^ according to Adult Treatment Panel III (ATPIII).

**Table 4:** Anthropometric features, ‘Junk Score’, and intestinal permeability according to adherence to Mediterranean Diet subgroups.

	Adequate Adherence Score ≥10	Inadequate Adherence Score <10
Number	93	27
Males (%): Females (%)	43 (46%):50 (54%)	16 (59%):11 (41%)
Age (years)	46.8 ± 1.4	38.5 ± 2.2 *
BMI (kg/m^2^)	27.7 ± 0.7	28.7 ± 1.1
Normal weight, prevalence (%)	38 (41%)	7 (26%)
Overweight, prevalence (%)	24 (26%)	6 (22%)
Obese, prevalence (%)	31 (33%)	14 (52%)
Waist circumference (cm) ^1^		
Males	99.2 ± 2	101.3 ± 2.5
Females	90.7 ± 2.5	85 ± 6.2
Waist circumference (cm) ^2^		
Males	101.7 ± 2.3	107.6 ± 3.2
Females	98.3 ± 2.6	92.4 ± 5.8
Hip circumference (cm)	105.5 ± 1.5	107.7 ± 2.3
Neck circumference (cm)		
Males	41.5 ± 0.6	42 ± 0.7
Females	35.8 ± 0.6	35.7 ± 1.27
‘Junk Score’	10.2 ± 0.5	13.8 ± 1 *
Stomach (sucrose recovery, %)	0.04 ± 0.002	0.04 ± 0.005
Small intestine (lactulose/mannitol, ratio)	0.02 ± 0.008	0.02 ± 0.002 *
Colon (sucralose recovery, %)	1.19 ± 0.06	1.23 ± 0.15

Data expressed as mean ± SEM. Differences tested by Mann–Whitney U test.Significance levels: * *p* < 0.05 vs. adequate adherence. ^1^ According to International Diabetes Federation (IDF); ^2^ according to Adult Treatment Panel III (ATPIII).

## Abbreviations

BMI	body mass index
IP	Intestinal permeability
LA	lactulose
MA	mannitol
NAFLD	non-alcoholic fatty liver disease
SA	sucralose
SIBO	small intestinal bacterial overgrowth
SO	sucrose
